# Pulmonary Embolism in a Woman Taking Oral Contraceptives and Valdecoxib

**DOI:** 10.1371/journal.pmed.0020197

**Published:** 2005-07-26

**Authors:** Elizabeth J Westgate, Garret A FitzGerald

**Affiliations:** 1Case report from the Institute for Translational Medicine and Therapeutics, University of Pennsylvania, Philadelphia, Pennsylvania, United States of America

## Abstract

A 25-y-old woman, who had been on an oral contraceptive pill for 3 years, presented with pulmonary embolism. One month prior to presentation she had been started on valdecoxib for neck pain.

## PRESENTATION of CASE

A 25-y-old woman presented with pulmonary embolism. She had been taking, without apparent complication, norgestimate/ethynil estradiol (Ortho Tri-Cyclen; 0.180, 0.215, and 0.250 mg norgestimate cycles and 35 μg ethinyl estradiol) for 2 y, followed by the same drug combination but with a lower dose of ethinyl estradiol (Ortho Tri-Cyclen Lo; 25 μg ethinyl estradiol) for 14 mo. A nonsmoker, she lacked a relevant family history and was vigorously athletic.

**Figure 1:**
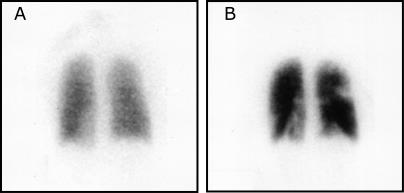
Ventilation-Perfusion Scan (A) After inhalation of 20.1 mCi of Xenon-133 gas, scintigraphic images were obtained in the posterior projection, showing uniform ventilation to lungs. (B) After intravenous injection of 4.1 mCi of Technetium-99m-labeled macroaggregated albumin, scintigraphic images were obtained, shown here in the posterior projection. This and other views showed decreased activity in the following regions: apical segment of right upper lobe, anterior segment of right upper lobe, superior segment of right lower lobe, posterior basal segment of right lower lobe, anteromedial basal segment of left lower lobe, and lateral basal segment of left lower lobe.

One month prior to presentation, she developed neck pain; disc protrusion at C5-C6 was detected by magnetic resonance imaging. The patient was prescribed valdecoxib, 20 mg twice a day (b.i.d.), for 2 wk. Her neck pain resolved. However, towards the end of this treatment period, she developed left-sided pleuritic chest and shoulder pain after a 6-h car ride. She was started on cyclobenzaprine, 10 mg b.i.d., and continued on valdecoxib. Her left-sided pain abated gradually. However, 18 d later, she developed right-sided chest and shoulder pain. A diagnosis of left iliac vein thrombosis and bilateral pulmonary emboli was based on a computed tomography scan. She was heparinized and continued on therapy with warfarin, 5 mg b.i.d., and enoxaparin, 60 mg b.i.d. Despite this, a ventilation-perfusion scan performed 13 d later showed multiple pulmonary emboli (Figure 1).

Currently, the patient is on warfarin, 2.5 mg daily, while completing a 6-mo warfarin regimen. Her warfarin is well tolerated and otherwise the patient is in good health.

## DISCUSSION

Coxibs, selective inhibitors of cyclooxygenase-2 (COX-2), increase the risk of myocardial infarction and stroke [1–3], prompting concern for patients with established cardiovascular disease. Caution may also extend to individuals predisposed to thrombosis by genetic or environmental factors.

Risk factors for spontaneous thrombosis include oral contraceptives, genetic predisposition to a hypercoaguable state, and prolonged periods of stasis [4–6]. At least two risk factors pertained to this patient.

The patient had been taking oral contraceptives for 3 y prior to the index event, albeit without recognized thrombotic complication. It is possible that despite this extended period of apparent tolerance, the embolic events were solely related to use of the oral contraceptives [4].

The relatively small risk of venous thromboembolism attributable to oral contraceptive use may interact geometrically with the similarly small absolute risk of a procoagulant mutation, such as Factor V Leiden [5]. However, documented genetic risk factors, such as abnormalities in lupus anticoagulant, anti-thrombin III, proteins C and S, plasma homocysteine, anticardiolipin, β2 glycoprotein antibody, and prothrombotic mutations in Factor V and prothrombin were excluded. It remains possible that the patient was genetically predisposed to thrombosis by a mutation in an undetermined factor.

Finally, prolonged stasis, such as that which occurred during the 6-h car trip, may account for the clinical event [6]. However, the absolute risk is small, and the patient had made this trip on multiple occasions devoid of apparent clinical complications during the prior 3 y.

The risk of thrombosis from valdecoxib has now been established [3,7], and this, together with its propensity rarely to cause Stevens-Johnson syndrome without a mitigating benefit over traditional nonsteroidal anti-inflammatory drugs, has led to its withdrawal from the market**.** The cardiovascular hazard of coxibs appears likely to be attributable to the suppression of COX-2-derived prostacyclin [1,8]. Deletion of the prostacyclin receptor in mice does not cause spontaneous thrombosis, but rather enhances the response to thrombotic stimuli [9]. This is consistent with the fact that a cardiovascular signal from a coxib is most easily detected in patients with hemostatic activation, such as was observed under placebo-controlled conditions in two trials in patients undergoing cardiopulmonary bypass grafting [7] and anecdotally in patients with connective tissue disease [10].

Although this case does not establish a causative linkage with valdecoxib, the clinical event was not manifest until three potential risk factors were combined (oral contraceptives, prolonged stasis, and coxib treatment). Given the multiplicative interactions of risk factors for thromboembolic disease and the apparently untoward concurrence of two of them—the contraceptive pill and frequent road trips for the preceding 3 y—the rapid occurrence of the clinical event following initiation of the coxib suggests a causative link to the COX-2 inhibitor. However, it also remains formally possible that this temporal relationship was a coincidence.

This case report has been reported to the regulatory authorities and to the manufacturer of valdecoxib.

Just as the small absolute risks of thrombosis attributable to oral contraceptives and prothrombotic mutations may interact dramatically [5], selective inhibitors of COX-2 may also interact with genetic and environmental factors that predispose to the risk of thrombosis.

**Learning Points**

• The risk of thrombotic events on COX-2 inhibitors may extend to apparently healthy individuals.

• Genetic and environmental risk factors for thrombosis may interact geometrically.

• Patients should be screened for recognized genetic or environmental predisposition to thrombosis prior to deciding to initiate treatment with COX-2 inhibitors.

## Abbreviations

b.i.d.: twice a day
COX-2: cyclooxygenase-2
